# Local intestinal microbiota response and systemic effects of feeding black soldier fly larvae to replace soybean meal in growing pigs

**DOI:** 10.1038/s41598-021-94604-8

**Published:** 2021-07-23

**Authors:** Soumya K. Kar, Dirkjan Schokker, Amy C. Harms, Leo Kruijt, Mari A. Smits, Alfons J. M. Jansman

**Affiliations:** 1 Animal Nutrition, Wageningen Livestock Research, Wageningen University & Research, Wageningen, The Netherlands; 2 Wageningen Livestock Research, Animal Breeding and Genomics, Wageningen University & Research, Wageningen, The Netherlands; 3 Netherlands Metabolomics Centre, Leiden University, Leiden, The Netherlands; 4 Department of Analytical Biosciences, Leiden University, Leiden, The Netherlands

**Keywords:** Animal physiology, Metabolomics, Microarray analysis, Microbiome

## Abstract

Black soldier fly (*Hermetia illucens*; BSF) larvae as dietary protein source have the ability to deliver nutrients and could possess functional properties that positively support animal productivity and health. More knowledge, however, is needed to assess the impact of feeding a BSF based diet on gut and animal health. Sixteen post-weaned male pigs were randomly assigned to two groups and fed for three weeks with iso-caloric and iso-proteinaceous experimental diets prepared with either soybean meal (SBM) as reference protein source or with BSF as single source of dietary protein. At the end of the trial, the pigs were sacrificed to collect relevant digesta, gut tissue and blood samples to study changes induced by the dietary treatments using ~ omics based analyses. Inclusion of BSF in the diet supports the development of the intestinal microbiome that could positively influence intestinal health. By amine metabolite analysis, we identified two metabolites i.e. sarcosine and methionine sulfoxide, in plasma that serve as markers for the ingestion of insect based ingredients. BSF seems to possess functional properties indicated by the appearance of alpha-aminobutyric acid and taurine in blood plasma of pigs that are known to induce health beneficial effects.

## Introduction

The search for sustainable alternatives has led to a growing interest in insects as an alternative protein source in diets for farm animals^1–4^. Insect meals as a protein source have the ability to deliver nutrients, particularly dietary amino acids (AA) and could provide functional properties, e.g. via the presence or release of bioactive substances with antimicrobial properties^5,6^, which positively support animal health and productivity. In addition, insect production has a small environmental footprint compared to the production of other dietary protein sources^7–9^. Among the insect species that are mass reared, is the black soldier fly *Hermetia illucens* (BSF). Black soldier fly has received considerable attention, because they can grow on different substrates, including organic waste streams^10^. Furthermore, BSF has the ability to convert organic waste into valuable nutrients and constituents for the host, particularly proteins, lipids, and bioactive substances^5,6,11^. Due to these characteristics, BSF has recently gained attention in pig farming as source of dietary protein^12,13^. However, an evaluation of the impact of this novel protein sources on the physiology and health of the animal is essential, before novel ingredients can be included in the diet.

**Figure 1:**
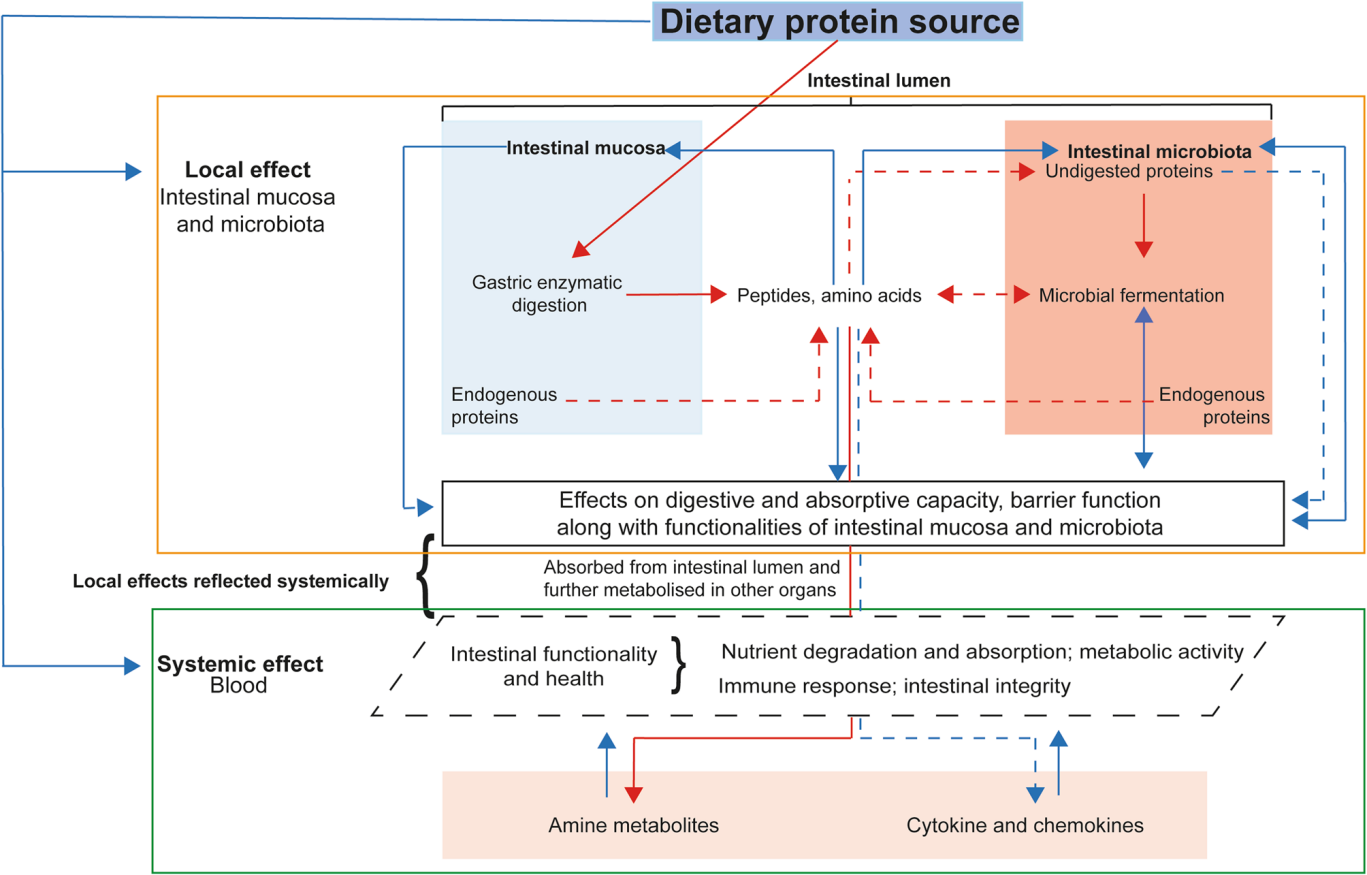
Schematic overview of effects of dietary protein sources on digestive physiology and systemic nutrient metabolism in pigs. Red arrows represent steps related to digestive physiology. Blue arrows relate to bio-functional effects of dietary protein sources and products released during their enzymatic hydrolysis. Continuous arrows represents major effects and dashed ones minor effects. The effects of dietary protein digestion at local (outlined in orange; black box, local effects) and systemic (outlined in green; dotted black box, local effects reflected systemically) levels.

It is known that the enzymatic hydrolysis and fermentation products formed during the passage of dietary proteins through the digestive tract can influence intestinal functionality and health (Fig. 1). Local effects can be recorded from analysis of digesta and intestinal tissue samples, whereas systemic effects of diets can be captured via analysis of blood samples. From studies in rodent models and humans, it is evident that high-throughput ~ omics based technologies could successfully capture the interaction of dietary protein sources with intestinal mucosal tissue and the resident microbiome^14–16^. Furthermore, measuring amine metabolites in blood is considered a useful approach to assess the metabolism of dietary protein by the host and the intestinal microbiome. On the one hand, the profile of amine metabolites in blood would reveal information about the nutritional value and utilization of protein sources and on the other hand about the metabolic and absorptive capacity of the gut, including the influence of the intestinal microbiome^17^. Systemic amine metabolic profiles are anticipated to be candidate biomarkers for dietary protein-associated phenotypes in relation to health^18,19^. Therefore, measuring or assessing the impact of interaction between dietary protein sources with the intestinal mucosal tissue and the resident microbiome along with the amine metabolite profiles in blood employing high-throughput ~ omics based technology is considered a useful approach to evaluate dietary protein sources^20,21^. In this context, “FeedOmics” provides an essential tool kit that combines multiple ~ omics techniques to evaluate dietary protein sources^22^. The FeedOmics approach can simultaneously gather comprehensive knowledge about (i) the molecular mechanisms involved in processing dietary protein in the digestive tract, diet induced changes in microbial communities in the gut and in host physiology, and (ii) functional properties of dietary protein sources in relation to gut health and functionality. By using ~ omics techniques in the FeedOmics approach one can generate relevant data that allows researchers to obtain a deep and thorough insight into biological responses of the animal towards differences in diet composition. However, such an approach has not been previously used in relation to diets containing BSF as a replacement for soybean meal (SBM) as a dietary protein source in growing pigs.

Thus, by enabling a FeedOmics approach, we evaluated the impact of diets formulated with either SBM as reference protein source or with BSF in growing pigs at the intestinal and systemic level. Briefly, we focused on the functionality of the small intestine by transcriptomics analysis on gut tissue and profiling the resident microbial community in both jejunum and ileum. Additionally, amine profiles in blood were analyzed to evaluate the impact of the diets at a systemic level. In addition, we measured cytokine and chemokine concentrations in blood as a reflection of the health and immune status of the pigs.

## Results

**Figure 2:**
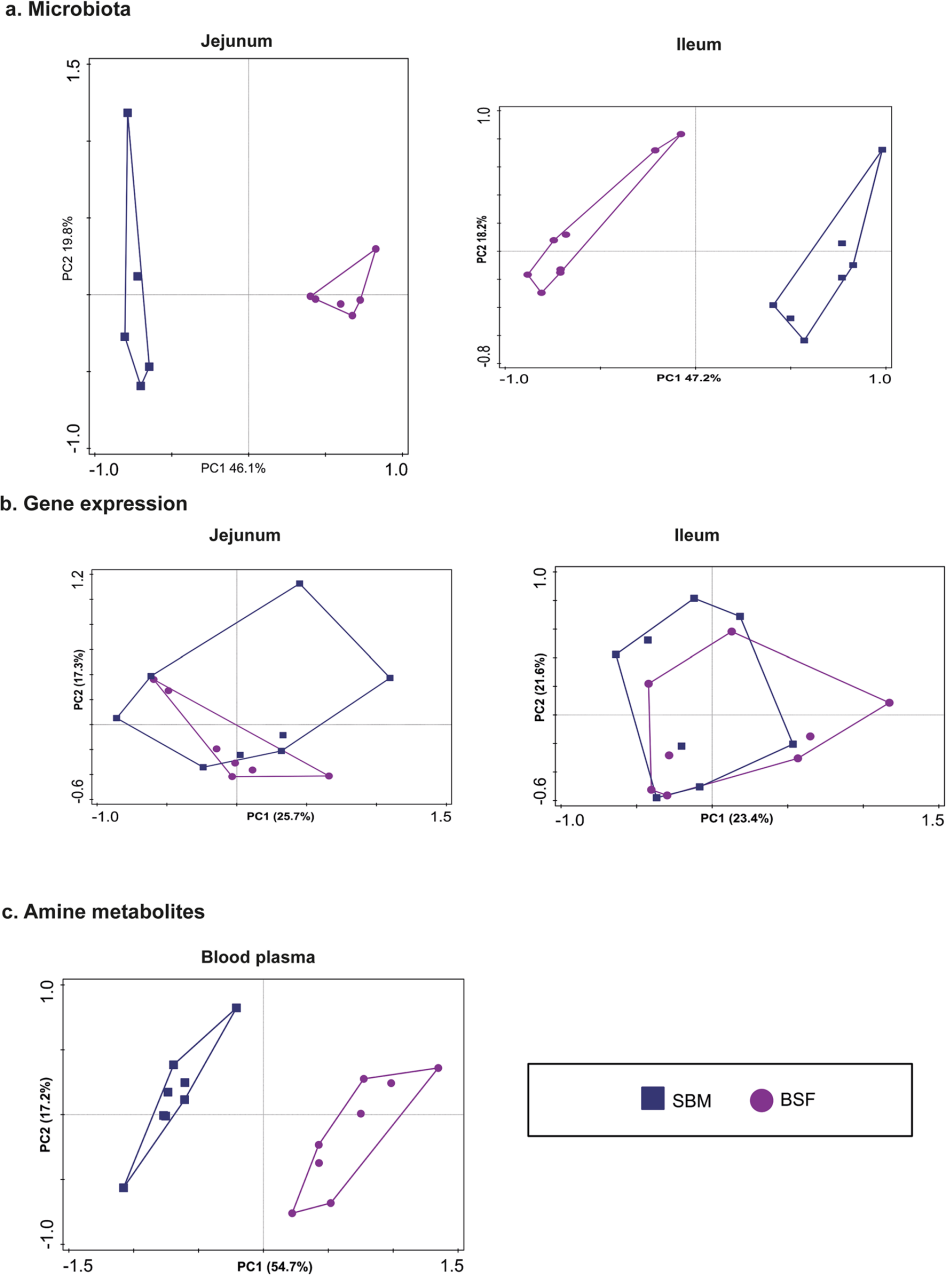
Response of pigs fed a diet with either soybean meal (SBM) or black soldier fly (BSF) as single protein source. Principal Component Analysis (PCA) plot explaining variation in the in (**a**) intestinal microbiota composition, (**b**) gene-expression in intestinal mucosal tissue, and (**c**) systemic amine metabolite profiles. The PCA displays 95% confidence regions for each dietary treatment.

Principle component analysis (PCA) was performed to get more insight into the variability of different responses measured by the ~ omics based technologies. The variance explained in the microbiome composition by the first two axes was 65.9% for jejunum and 65.4% for ileum (Fig. 2a). In addition, the variance explained in the genome-wide transcriptomics responses by the first two axes was 43.0% for jejunum and 45.0% for ileum (Fig. 2b). Based on the variance explained by the first two axes, dietary treatment explained the highest proportion of variance i.e. 71.9% for amine metabolites in blood plasma (Fig. 2c).The PCA plots of the amine metabolites and the microbiota composition displayed clear clustering of pigs fed either the SBM or the BSF based diet. Notably, the PCA plot of gene expression in jejunum and ileum tissue displayed an overlap between dietary treatment groups.

### Microbiota response

#### Small intestinal microbiota response

Illumina Mi-Seq sequencing of the 16S rRNA (V3 region) amplicons generated more than 14.9 million high-quality reads that were classified into OTUs with an average of 576,290 ± 161,261 (SD) reads per sample (Supplementary Table S1). The average number of OTUs per sample after filtering was 1,091 ± 323. The relative abundance of the identified microbiome is presented in Supplementary file S1.

To characterize the phylogenetic composition of bacterial communities in different small intestinal locations as affected by the dietary treatments, we first performed the 16S rRNA gene sequencing analysis and compared the alpha-diversity of microbiota among jejunal and ileal digesta samples of pigs fed the BSF or SBM based diet. Feeding the BSF diet resulted in a significantly higher Shannon index for the microbiota in the jejunum whilst no difference was observed for the microbiota in the ileum (Table 1). We then explored the variation of phylogenetic diversity among all digesta samples collected from both jejunum and ileum. All samples clustered more distinctively by diet compared to the location in the Principal Coordinate Analysis (PCoA) plot (Supplementary figure S1). The digesta samples from the jejunum and ileum of pigs fed BSF or SBM based diet had a significantly distinct phylogenetic diversity when comparing both small intestinal locations (P < 0.01; PERMANOVA test; Table 1).

**Table 1:** Microbiota composition and diversity in jejunum and ileum digesta of pigs fed a diet with either soybean meal (SBM) or black soldier fly (BSF) as single protein source.

Tissue	Jejunum			Ileum		
Group	SBM	BSF	P-value	SBM	BSF	P-value
**Alpha diversity**						
*Shannon index*	3.04 ± 0.30	3.76 ± 0.13	**0.03**	2.87 ± 0.25	3.46 ± 0.16	0.18
**Beta diversity**						
Comparisons	BSF vs SBM			BSF vs SBM		
	P-value	P-value (Bonferroni)		P-value	P-value (Bonferroni)	
PERMANOVA analysis: *Bray Curtis*	**0.0002**	**0.0009**		**0.002**	**0.01**	

**Figure 3:**
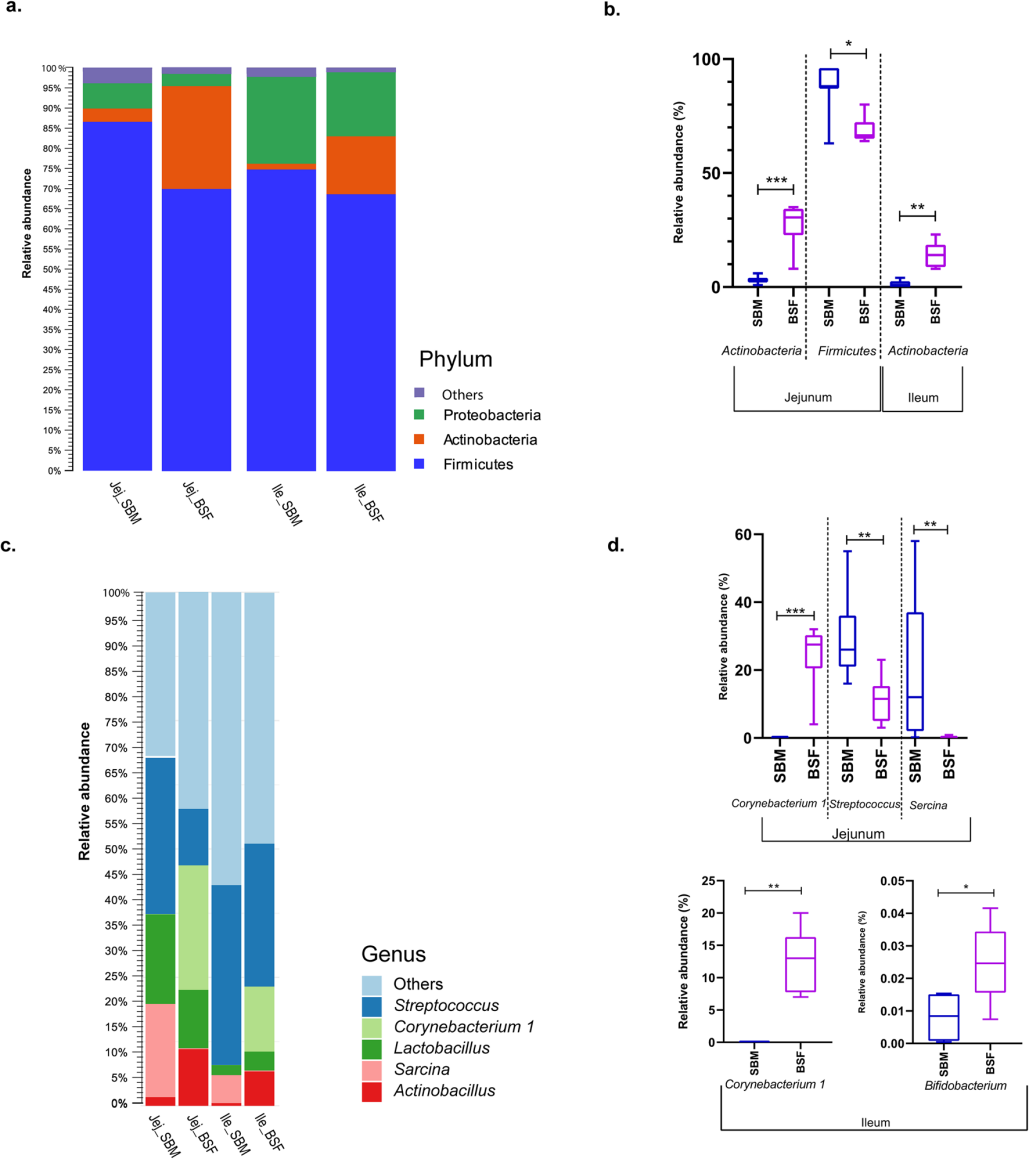
Bacterial abundances in digesta from jejunum and ileum of pigs fed a diet with either soybean meal (SBM) or black soldier fly (BSF) as single protein source. (**a**) Top three bacteria classified at phylum level. (**b**) Relative abundance of amongst the top three bacteria at phylum level that differ significantly between digesta collected from jejunum and ileum of pigs fed SBM and BSF based diet. (**c**) Top five bacteria classified at genus level. (**d**) Relative abundance of amongst the top five bacteria at genus level along with the relative abundance of *Bifidobacterium* that differed significantly among digesta collected from jejunum and ileum of pigs fed the SBM or BSF based diet. *** P value < 0.0005, ** P value < 0.005, * P value < 0.05.

Bacteria classified among the *Firmicutes*, *Proteobacteria* and *Actinobacteria* phyla were the three most abundant in digesta collected from both the jejunum and the ileum (Fig. 3a). In the digesta samples of pigs fed BSF, the top three phyla accounted for 98.9% and 99.2% of the relative abundance in the jejunum and the ileum, respectively, whereas, the relative abundance of the top three phyla accounted for 96.4% for digesta in the jejunum and 97.7% for digesta in the ileum of pigs fed the SBM diet. Among the observed top three phyla, the relative abundance of *Firmicutes* was numerically the highest in the jejunum and ileum, accounting 68.8% for BSF, 86.7% for SBM in jejunum and 69.2% for BSF and 73.0% for SBM in ileum. Comparing both dietary treatment groups for the relative abundance in digesta from jejunum and ileum for the *Firmicutes*, a significantly lower relative abundance was observed for BSF fed pigs in the jejunum (Fig. 3b). The relative abundance of *Proteobacteria* accounted 2.6% for BSF and 6.4% for SBM in jejunum, whereas 15.8% for BSF and 23.4% for SBM in the ileum, although no significant differences were observed. In addition, we noticed a significantly higher relative abundance of *Actinobacteria* phyla in the jejunum of pigs fed BSF (i.e. 27.5%) compared to SBM fed pigs (i.e. 3.3%). A similar significantly higher relative abundance of *Actinobacteria* phyla was observed in the ileum of pigs fed BSF (14.2%) compared to SBM fed pigs (1.3%; Fig. 3b).

At genus level, bacteria classified among the *Streptococcus*, *Lactobacillus*, *Sarcina* within the phylum *Firmicutes*, *Corynebacterium 1* within the phylum *Actinobacteria*, and *Actinobacillus* within the phylum *Proteobacteria* were the five top most abundant genera in digesta collected from both jejunum and ileum (Fig. 3c). The top five genera accounted for 58% and 52% of relative abundance in the jejunum and the ileum, respectively. Among the observed top five species, the relative abundance of *Corynebacterium 1*, *Streptococcus* and *Sarcina* differed significantly between the treatment groups in jejunum (P < 0.05) and only the relative abundance of *Corynebacterium 1* differed significantly between dietary treatment groups in the ileum (Fig. 3d). A significantly higher abundance of the genus *Bifidobacterium* was observed in ileum digesta of pigs fed the BSF diet than in pigs fed the SBM diet (Fig. 3d).

Differential abundance analysis (DAA) of the microbiota composition in digesta of both treatments identified 65 and 53 significant differential abundant genera (FDR < 0.05 with fold change > 5) in the jejunum and then ileum, respectively (Supplementary file S1). Redundancy analysis of treatment effects also revealed a significant impact on microbiota composition, with discriminating microbial genera such as *Corynebacterium 1, Globicatella, Oceanobacillus and Bacillus* associated with the BSF diet and *Phaseolus acutifolius, Lotus japonicus, Citrus maxima* associated with the SBM diet in both the jejunum and the ileum (Supplementary figure S2; Supplementary file S1). Both intestinal locations illustrate a high level of congruency for the BSF-associated genera (Supplementary file S1), indicating that diet effect is greater than the location effect.

#### Small intestine mucosal whole-genome transcriptome response

A low variability within treatment group in gene expression in the intestinal mucosa was displayed in the PCA plot (Fig. 2b). No genes were significantly differentially regulated (P < 0.05 and FC > onefold or FC <  − onefold) between treatment groups in both the jejunal and ileal tissue. Further analysis with gene set enrichment analysis (GSEA) revealed multiple core-enriched genes (6 to 118 genes) that are involved or part of differential enriched gene sets (FDR < 0.1; Supplementary Table S2). Briefly, compared to the samples of pigs that received the SBM diet, upregulated pathways were found to be mostly associated with tryptophan metabolism and peroxisome proliferator-activated receptor (PPAR) signaling pathways in the ileal mucosa of pigs that received the BSF diet. Downregulated pathways were associated with neuron differentiation, neuron development, and cell–cell adhesion. Details of the core enriched genes in biological processes and pathways are summarized in Supplementary file S2.

#### Post-absorptive plasma amine metabolomic responses

**Figure 4:**
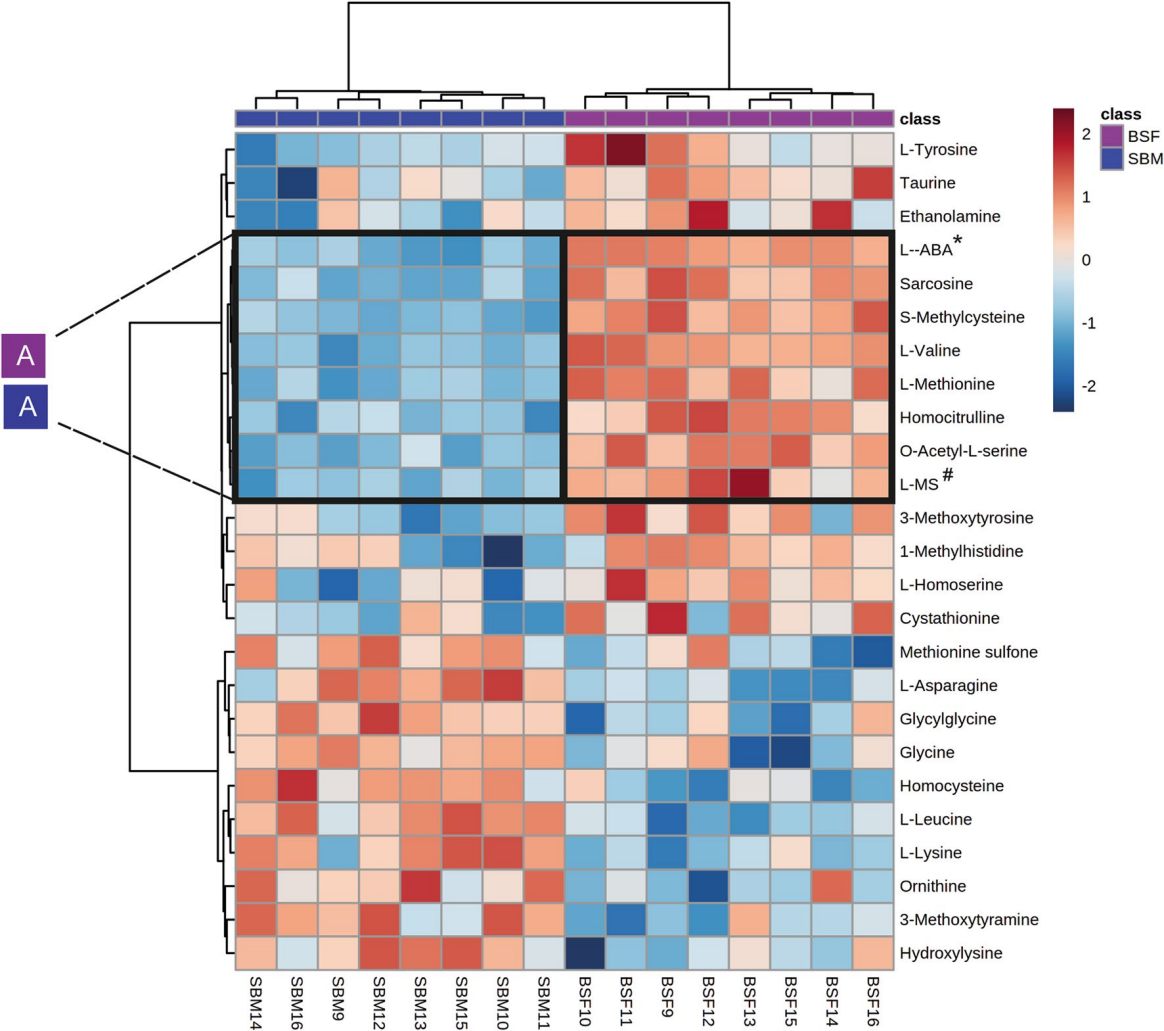
Heat-map of clustering the plasma amine metabolites of pigs in dependence of dietary treatment. SBM: Soybean meal; BSF: Black soldier fly. Values are the relative response ratios of each metabolite of an individual pig per treatment group. Treatment groups comprise of eight pigs. Values are measured in plasma of pigs at d 28 of the experimental period. The heat map graphic distances were measured using Euclidean distances and the clustering algorithm using ward dendrogram. Each colored cell on the map corresponds to a concentration value (normalized to the SBM treatment). The alphabets “A” represent the diets associated cluster formed by set of metabolites (framed in black) having low (shades of blue) or high (shades of red) concentration in the dietary treatments. Here, the colored square corresponds to the respective experimental diets; i.e. navy blue for SBM, and magenta for BSF. “A” indicates amine metabolite clusters referred to the text. *L-ABA: L-Alpha-aminobutyric acid and ^#^L-Methionine sulfoxide.

**Figure 5:**
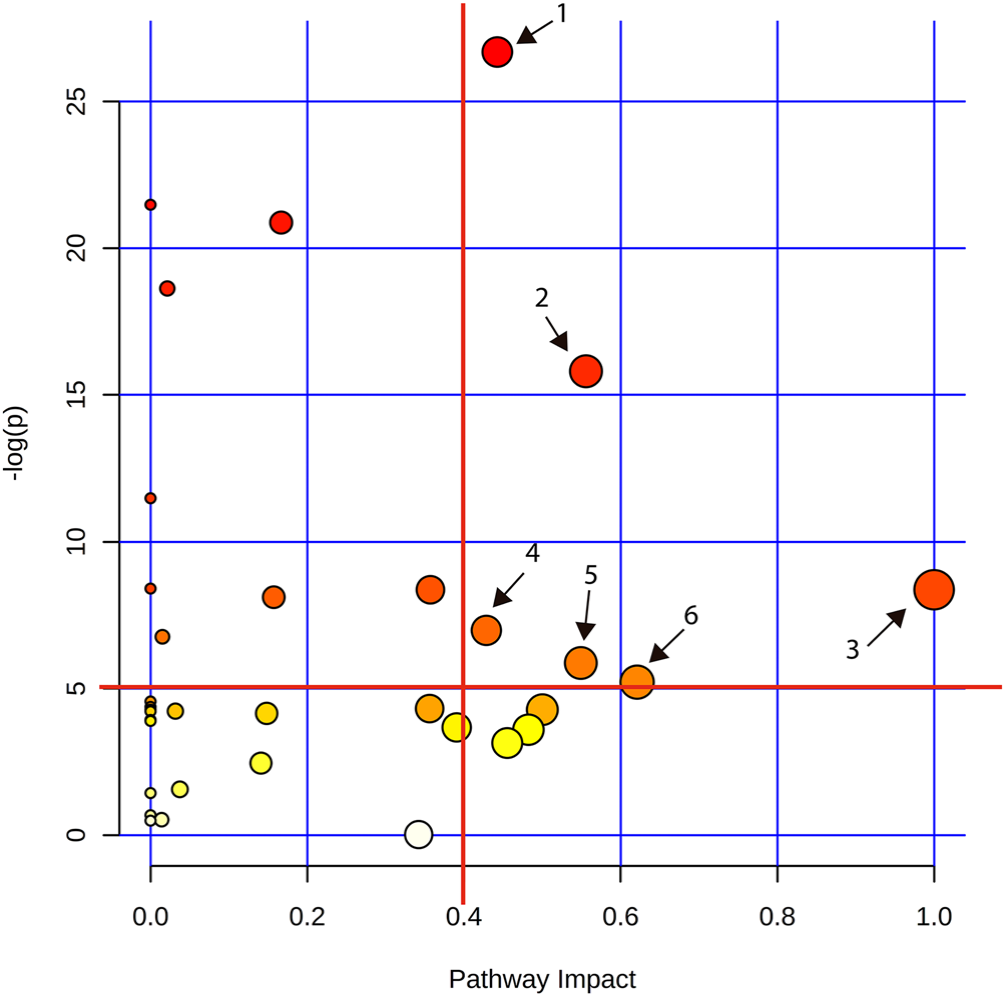
Metabolic pathway analysis based on plasma amine profiles in pigs fed a BSF or SBM based diet. The graph represents the result of the metabolic pathway analysis. Scores from the topology analysis on the ‘x axis’ and from the enrichment analysis are represented on the ‘y axis’. The size and the color (on gradient scale: white to red) of the nodes are all matched pathways according to the p values from the pathway enrichment analysis and pathway impact values from the pathway topology analysis. The red lines denote the thresholds in both axes to identify the most significant matched pathways for all the dietary comparisons. The arrow indicates to the enriched pathways, where, 1 is cysteine and methionine metabolism; 2 is glycine, serine and threonine metabolism; 3 is phenylalanine, tyrosine and tryptophan biosynthesis; 4 is taurine and hypotaurine metabolism; 5 is histidine metabolism and 6 is alanine, aspartate and glutamate metabolism.

The heatmap revealed a diet-associated metabolic-set, i.e. cluster A (Fig. 4). Cluster A comprises of L-alpha-aminobutyric acid, sarcosine, S-methylcysteine, L-valine, L-methionine, homocitrulline, O-acetyl-L-serine and L-methionine sulfoxide which were found at relatively high concentrations in plasma of BSF-fed pigs and in low concentrations in the SBM fed pigs (Supplementary Table S3). Plasma amine metabolites which differed in concentration between SBM and BSF treatment groups were mapped to the Kyoto Encyclopedia of Genes and Genomes (KEGG) metabolic pathways. This revealed six metabolic pathways that were influenced by the dietary treatment (Fig. 5). The enriched KEGG pathways were cysteine and methionine metabolism, glycine, serine and threonine metabolism, phenylalanine, tyrosine and tryptophan biosynthesis, taurine and hypotaurine metabolism, histidine metabolism and alanine, aspartate and glutamate metabolism. In these pathways, differences between BSF and SBM groups were observed in the concentration of the amine metabolites (Table 2).

**Table 2:** Metabolic pathways involving amine metabolites differently expressed in pigs receiving a BSF or SBM based diet.

	Enriched pathways	Pathway analysis				Pathway match status		Metabolites among the enriched pathway that differ significantly (P < 0.05)				
		Pathway enrichment analysis (Raw P value)	Pathway enrichment analysis (FDR)	Pathway topology analysis: pathway impact		Total number of metabolites in pathway	Number of metabolite hits within a pathway	Number of metabolites	Metabolites	Pathway impact	P value	Differential abundant in BSF compared to SBM
**1**	Cysteine and methionine metabolism	2.57E-12	8.74E-11	0.44255		33	5	3	L-methionine	0.1	5.60E-09	High
									L-cystathionine	0.18	0.00001	High
									(s)-2-aminobutanoate	0.04	5.71E-10	High
**2**	Glycine, serine and threonine metabolism	1.37E-07	9.33E-07	0.55577		33	6	2	L-cystathionine	0	1.00E-04	High
									Sarcosine	0.09	6.67E-08	High
**3**	Phenylalanine, tyrosine and tryptophan biosynthesis	0.000232	0.000878	1.00		4	2	2	L-phenylalanine	0.5	0.007	High
									L-tyrosine	0.5	0.0002	High
**4**	Taurine and hypotaurine metabolism	0.000928	0.002869	0.42857		8	2	1	Taurine	0.4	0.001	High
**5**	Histidine metabolism	0.002821	0.007377	0.54917		16	7	3	Methyl-L-histidine	0	0.0007	High
									Histamine	0.19	0.007	High
									L-glutamate	0	0.01	High
**6**	Alanine, aspartate and glutamate metabolism	0.005416	0.013154	0.621		28	6	3	L-alanine	0	0.01	High
									L-glutamate	0.2	0.01	High
									L-asparagine	0	0.02	Low

#### Systemic inflammatory marker response

Out of the nine cytokines and chemokines evaluated, only two (i.e. INF-α and IL-12p40) were consistently detected above the minimum detection limit in serum of six pigs per treatment collected at the start and at the end of the feeding trial. Values did not significantly differ between treatments (Supplementary figure S3).

## Discussion

Here, we adopted the FeedOmics approach that uses a set of ~ omics based techniques that can measure the impact of dietary protein sources on the physiology of the host. Data on the presence of microbial DNA, on the host transcriptomics for RNA and on the metabolomics for amine metabolites measured at different biological levels i.e. local (small intestinal) and systemic level provided a detailed and thorough snapshot of the response in pigs towards dietary protein source. Inclusion of BSF in the diet resulted in a more enriched small intestinal microbiome compared to feeding a SBM based diet. Our results indicate that BSF supports the growth of microbial taxa that are either indicators of a healthy gut or recognized as beneficial microbes that exert positive health effects in pigs. The small intestinal tissue functioning measured by whole-genome transcriptome profile responded in a less divergent manner but recorded subtle differences between both groups. Amine metabolite profiles in blood plasma showed the ability of BSF to provide functional properties that can be important for health and performance in pigs beyond their capacity to provide AA. Below, we discuss the impact of feeding BSF to replace soybean meal in growing pigs by measuring the responses in the gut and systemic effects.

The small intestinal microbiome data revealed that, irrespective of the dietary treatment, Firmicutes, Proteobacteria and Actinobacteria were detected as the three most abundant phyla in jejunal and ileal digesta of pigs. This is in agreement with previous studies^23–28^ which observed Firmicutes, Actinobacteria as the predominant phyla in pigs, suggesting that these phyla are core members of porcine intestinal microbiota^24^.

Striking differences between groups of pigs fed either the BSF or the SBM diet were revealed by the small intestinal microbiome data. We observed similar diet-specific microbial groups associated with the jejunum and ileum, thereby indicating that dietary treatment overrules the intestinal location-specific variation in small intestinal microbiota composition^29^. Compared to the SBM based diet, we observed a significant impact of the BSF based diet on the small intestinal microbiota composition, reflected by an increased microbial diversity (Shannon index, jejunum) and a significantly altered microbial abundance, both at phylum and genus level. It implies that, inclusion of BSF in pig diet, contributes to an increase in the richness of the intestinal microbiota diversity which is crucial and protective for sustaining a microbial equilibrium, a stable composition of species within the microbiome, and for the integrity of the mucosal barrier^30^. Overall, the inclusion of BSF in the diet resulted in a more enriched small intestinal microbiome compared to feeding a SBM diet. This is generally regarded as an indicator of a “healthy gut” in pigs^31^. Further, we observed significantly higher abundance of phylum Actinobacteria in pigs fed the BSF diet. Notably, various studies have previously reported an association of an increased abundance of the phylum *Actinobacteria* with “healthy pigs” compared to either diarrheic^32^ or antibiotic-treated pigs^24,33^.

Inclusion of BSF in the pig diet resulted in a significant increase of *Bifidobacterium* that belongs to the Actinobacteria phylum, in the ileum of pigs. *Bifidobacterium* is a genus known to impart positive health effects in humans^34^ and animals including pigs^35,36^. Several members of this genus have been widely recognized as “beneficial” microbes (probiotics) that offer positive effects to the host mainly by maintaining and supporting intestinal health and functionality^37,38^. Further analysis at genus level revealed that the BSF diet had a significant effect on microbiota composition in both jejunum and ileum. The BSF diet significantly increased genera such as *Corynebacterium 1*, *Globicatella*, *Oceanobacillus*, and *Bacillus* in the small intestine. *Corynebacterium 1* emerged as one of the predominant genera associated with the BSF diet in the small intestine of pigs.

Interestingly, a decreased abundance of *Streptococcus* was observed in our study that was also reduced in an in vitro study assessing the antimicrobial effect of a BSF based diet^5^. Additionally, we observed an increased abundance of *Corynebacterium* and *Globicatella*, which is similar to the findings of another study which evaluated piglets exposed to antibiotic treatment^33^. These findings suggest an effect of the BSF-based diet on the microbial community in the small intestine of pigs indicating that BSF possesses anti-microbial properties. The evidence of BSF having anti-microbial properties has been reported in literature^5,39,40^. The presence of lauric acid (C12:0), medium chain fatty acids^5^ or antimicrobial peptides^39,40^ in BSF has been attributed to the antimicrobial properties. In the present study, the pigs were fed defatted BSF suggesting that the observed suppressed bacterial growth or the matching microbiome abundance for the BSF based diet with an antibiotic treatment might be related to the presence of short and medium chain fatty acids and/or bioactive peptides having antimicrobial properties. This observation still needs to be verified in future research. Overall, our results on the small intestinal microbiome composition indicate that inclusion of BSF in pig diet supports the development of an intestinal microbiome that supports intestinal health and provides antimicrobial compounds, compared to the use of conventional protein sources i.e. SBM in the diet.

The small intestinal transcriptomics data revealed no striking differences between the dietary treatments, even with a less stringent filter settings applied for identifying differentially regulated genes, i.e. P < 0.05 and FC > onefold or FC <  − onefold. However, to gain further insight into the biological meaning of BSF-induced changes in the small intestinal transcriptome, we employed GSEA to identify subtle differences in transcriptome response. Only a more lenient significance level i.e. FDR < 0.1, in contrast to FDR < 0.05 as reported in^20^, enabled us to observe a few differentially enriched gene-sets (biological processes and pathways). Therefore, these results need to be carefully interpreted considering GSEA was not performed with differentially regulated genes and a lenient filter setting was applied for the significance level i.e. FDR < 0.1 in the GSEA. The BSF-based diet seemed to modulate genes involved in biological processes and pathways that are known to maintain or influence intestinal health or functionality. Use of a BSF diet in pigs up-regulated the expression of genes related to the peroxisome proliferator-activated receptor (PPAR) signaling pathway, particularly in ileal tissue compared to pigs fed the SBM based diet. The PPAR signaling pathway is known to be involved in the homeostatic regulation of cellular proliferation/differentiation and modulation of the inflammatory response in enterocytes^41,42^. Activation of the PPAR signaling pathway, particularly by conjugated linoleic acids (CLA) has been suggested to exert beneficial effects on intestinal health in pigs ^43^. Studies have confirmed the presence of CLA in BSF larvae and pupae^44–46^. Thus, being a source for CLA, BSF appears to regulate the PPAR signaling pathway. It demonstrates the capacity of BSF diet to impact the homeostatic regulation of proliferation/differentiation and modulation of inflammatory responses in non-challenged conditions in ileal tissue of pigs fed a BSF compared to a SBM based diet. In addition to intestinal immunity and metabolism, feeding a BSF-based diet appeared to regulate the intestinal cell barrier function, which was more pronounced in the jejunum compared to the ileum. In jejunum we observed that the BSF diet influenced several genes that are known to regulate the epithelial barrier function such as genes related to functioning of the tight junctions i.e. of the claudins protein family (CLDN7, CLDN10, CLDN2, CLDN8, CADM3, CLDN9, CLDN5, CLDN19) and genes acting as neuronal mediator i.e. neuropeptide Y (NPY gene)^47,48^. However, these findings warrant further validation in studies using quantitative polymerase chain reaction (qPCR) based methods to further quantify responses of selective genes.

It is known that intestinal microbiota interact with the host and therefore influence the small intestinal transcriptome responses^49^. It was assumed that differences in intestinal microbiota composition causes differences in host tissue intestinal gene expression or vice versa^50^. However, by only measuring the microbial diversity and composition without measurement of functionality of the microbiome does not allow us to establish one-on-one relationships with changes observed in the host gene expression. Such research is warranted.

An amine-based endophenotype concept was used to evaluate effects of dietary protein source^21^. Products of hydrolysis of dietary protein in the digestive tract and other N-containing constituents present in the diet which are absorbed from the digestive tract, enter the blood circulation and can be further used and metabolized in organs and tissues. The amine profile in blood plasma also provides a snap-shot view of dietary protein impacting the host-microbe interactions. We identified and measured amine profiles that consist of essential and non-essential AA, amine intermediate metabolites, which are important for cellular protein synthesis but also for biosynthesis of neurotransmitters (e.g. serotonin) and immunomodulatory metabolites (e.g. kynurenine), amines generated by microbial metabolism (e.g. putrescine) and precursors for other nitrogen based biologically active motifs (e.g. glutathione) in the blood plasma of pigs.

From the metabolic pathway analysis, we observed that dietary inclusion of BSF or SBM impacted metabolic pathways as expressed by a differential abundance of amine metabolites. These observations are in agreement with previous findings where pigs fed with insect meal from *Tenebrio molitor* larvae^51^ and mice fed with diets including different protein sources among which insect meal from *Tenebrio molitor*^21^. The enriched metabolic pathways are mainly related to AA metabolism. Within the enriched metabolic pathways, the abundance of AA and other amine metabolites were recorded higher in pigs fed the BSF diet compared to the SBM diet. In particular, the concentration of AA such as phenylalanine and tyrosine was significantly (P < 0.05) higher in plasma of pigs fed the BSF diet, in line with the difference in dietary concentration on an apparent ileal digestible basis. Further, based upon combined results of the heat-map and metabolic pathway analysis, methionine and valine were recorded high in the plasma of pigs fed the BSF diet compared to the SBM diet. The observed differences in AA metabolism are probably in part related to differences in the amount of AA provided via the diet, as the calculated AA composition on ileal digestible basis was different for most AA, and to their supply relative to the AA requirements of pigs for maximum growth performance. It should be noted that the diets in the present study were not formulated to be balanced for essential amino acids relative to the assumed AA requirements of pigs. A higher concentration of sarcosine was observed in the plasma of pigs fed with BSF. Both sarcosine and methionine sulfoxide were found highly discriminative among the amine-based endophenotypes in blood plasma of pigs fed the BSF diet or the SBM diet. Insects and products derived of were previously shown to contain sarcosine and methionine sulfoxide^52^. Furthermore, a relatively high concentration level of methionine sulfoxide was also reported in plasma of growing pigs fed insect meal from *Tenebrio molitor*^51^. Thus it was confirmed that increase of plasma methionine sulfoxide concentration is an insect meal-specific effect in pigs fed either *Tenebrio molitor-* or BSF-based diets.

Moreover, in addition to being a good source of AA as part of the “strict-nutritional” value BSF, compared to SBM, has the capacity to deliver other “non-strict-nutritional,” functional properties characterized mainly by the appearance of alpha-aminobutyric acid and taurine in plasma of BSF fed pigs. Alpha-aminobutyric acid is a key intermediate in the synthesis of a tripeptide analog of glutathione (i.e., ophthalmic acid) with antioxidative properties that can support health of pigs^53^. Taurine (β-aminoethanesulfonic acid) is an AA derivative, present in various tissues of different insect species^54,55^, including BSF^56^. Taurine can exert health benefit effects in pigs by providing protective effects via regulating immune responses and restoring tight junctions in the gut mucosa when piglets suffer from oxidative stress^57,58^. Taurine supplementation is also proposed to be a potential nutritional intervention strategy to increase growth performance in pigs^59^.

Taken together, BSF has a high capacity to deliver both essential and non-essential AA, that are vital precursors for protein synthesis in growing animals and the synthesis of organic nitrogen containing compounds, affecting metabolic processes and health. Feeding a BSF based diet to pigs resulted in elevation of sarcosine and methionine sulfoxide in plasma that serve as a markers for the presence of insects in the diet. Additionally, by adopting amine-based endophenotype approach, we could evaluate functional properties of BSF, indicated by the appearance of alpha-aminobutyric acid and taurine in plasma of BSF fed pigs.

To investigate whether the BSF-based diet induced changes in systemic immunity we measured a panel of cytokines and chemokines in the serum of the pigs. A study in mice showed that that dietary protein sources can alter systemic cytokines and chemokines responses^20^. However, investigations on the effects of feeding BSF as the only protein source towards the inflammatory markers in blood of pigs are lacking. In this study, we recorded for the first time the effects of BSF on a penal of nine inflammatory markers in blood of pigs by measuring the levels of pro- and anti-inflammatory cytokines and chemokines (i.e. IFNα, IFNγ, IL-1β, IL-10, IL-12p40, IL-4, IL-6, IL-8, and TNFα). The findings showed that the systemic cytokines and chemokines were mostly unaffected and no significant (P < 0.05) differences were observed for cytokines that could be detected i.e. INF-α and IL-12p40 between dietary treatments. This was expected as the experimental pigs were apparently healthy with no records of clinical signs of disease. Furthermore, these observations were in line with results of the small intestinal transcriptome analysis in which we could not observe signs of small intestinal inflammation in animals subject to either of the dietary treatments.

In this study, insect meal from BSF was included in the diets as only source of protein to replace SBM on an iso-nitrogenous and isoenergetic basis, while not fully balancing the diets for the level of essential AA. This could have affected the growth performance of the pigs. No significant (P < 0.05) difference was observed for average body weight of pigs fed either a BSF- or SBM-based diet. This observation should not be overstated as the present trial was not set up as a performance study. Such a trial would require a higher number of animals per treatment. However, the result of the present study is in line with results of other studies in which dietary inclusion of BSF replacing SBM did not affect growth performance of pigs^60–62^.

In conclusion, the functional value of BSF as dietary protein source was revealed by a FeedOmics approach that showed effects on the small intestinal microbiome and the profile of blood plasma amine metabolites. Such functional value could ultimately improve the competitiveness and the economic perspective of insect meals as sustainable feedstuffs for pig diets compared to conventional protein sources. In addition, compared to feeding a SBM-based diet, we observed no significant effects of dietary inclusion of BSF on the growth performance and on plasma cytokine and chemokine concentrations under non-challenge conditions. It is reasonable to conclude that BSF can be used as protein source in diets for growing pigs and partly replace SBM.

## Material and methods

### Protein sources and experimental diets

The protein sources evaluated were soybean meal (SBM; all commodity batches obtained via Research Diet Services, Wijk bij Duurstede, the Netherlands), and black soldier fly larvae meal (BSF) (obtained from Protix, Dongen, the Netherlands). The experimental diets were formulated to be iso-proteinaceous (CP, 160 g/kg as-fed basis) and included the respective protein sources as the only protein containing ingredients. Free DL-methionine and L-tryptophan were included to prevent severe limitations for these AA relative to the requirement values for these AAs^63^. For BSF, information on the AA profile and apparent ileal digestibility of AA was obtained from literature^64,65^. Titanium dioxide was included, at 2.5 g/kg feed (as-fed basis), in all the diets as a marker for measurement of nutrient digestibility. All diets were produced by Research Diet Services (Wijk bij Duurstede, the Netherlands).

### Animals and housing

All procedures were approved by the animal experimentation board at Wageningen University & Research Center (accession number 2014099.b). A schematic representation and detailed description of the experimental design^22^ along with the details of biological replicate per treatment is being provided in Supplementary figure S5. A total of 16 growing pigs (boars) (Topigs 20 × Tempo from Van Beek, Lelystad, the Netherlands) with an average initial body weight of 34.9 ± 3.4 kg on the day (d) of arrival were included in this study. Pigs were blocked on litter and pigs within a block were randomly allocated to one of the experimental diets with eight pigs per experimental diet. The pigs were housed individually in metabolic cages (1.3 × 1.3 m or 2.0 × 1.0 m) with a tender foot floor. The ambient temperature was kept at 24 °C on d 1 and 2, at 23 °C on d 3, and constant at 22 °C from d 4 and onwards. During d 1 to 27, the lights were turned on between 5.30 h till 19.00 h. During d 28 to 30, the lights were turned on between 2.30 h till 19.00 h. Body weights of animals were measured every week throughout the experimental period. To find statistical significance we performed Student’s T-tests on the average body weight of pig in each treatment groups (Supplementary figure S4).

### Feeding

During d1 to 6, pigs were fed SBM based commercial diet and gradually adapted to the experimental diets (Table 3). From d7 and onwards, pigs were fed only the experimental diets. The experimental diets were provided in a mash form and mixed with water at a ratio of 1:2. Water consumption was limited and an extra 0.3 l of water was provided after each feeding. The feeding level was 2.5 times net energy (NE) requirement for maintenance (293 kJ NE/kg BW°^.^^75^). During d7 to 26, the feed allowance was divided into two equal amounts, fed at 8.00 h and 16.00 h. During d27 to dissection days i.e. (d28-29), the feed allowance was divided into 6 equal amounts, fed starting at 5.30 h at intervals of 3 h.

**Table 3:** Ingredient and calculated or analysed^1^ nutrient composition of the experimental diets for pigs, as fed basis.

Diet formulation	Diet^2^		Composition continued	Diet^2^	
Item	SBM	BSF		SBM	BSF
**Ingredients, g/kg**			**Amino acids, g/kg**	**AID**^6^	**AID**
Maize starch	376	451	Lys	8.7	6.6
Sugar	100	100	Met	2.3	1.9
Dextrose	50	50	Cys	1.9	0.9
Cellulose^3^	50	50	Met + Cys	4.2	2.8
Soybean oil	43.3	6.4	Thr	5.1	4.7
Chalk	14.4	0	Trp	1.8	1.7
Mono sodium phosphate	10.1	11.7	Ile	6.3	5.1
NaCl	4.1	0	Arg	11.1	4.9
Sodium bicarbonate	1.4	6.7	Phe	7.3	10.1
Calcium carbonate	0	5	His	3.8	4.0
Calcium chloride	0	4.8	Leu	10.5	8.0
Premix	5	5	Tyr	5.1	8.0
Titanium di-oxide	2.5	2.5	Val	6.4	7.2
DL-Methionine	0.3	1.3			
L-Tryptophan	0	0.6			
Black soldier fly (larvae)	0	305			
Soybean meal	343	0			
*Total*	*1000*	*1000*			
**Composition**^**4**^**, g/kg**					
Dry matter^1^	904	919			
Crude protein^1^	166	158			
Sugars^1^	175	150			
Starch^1^	296	385			
NSP^5^	130	115			
Fat^1^	31	45			
Ash^1^	53	48			
Ca	8.2	11.8			
Gross energy, MJ/kg	10.7	10.7			

^1^Analysed nutrient compositions.

^2^Diet: SBM is soybean meal, and BSF is black soldier fly larvae meal.

^3^Arbocell (JRS, Germany).

^4^Diets composition were formulated using data on ingredient nutrient composition and nutrient digestibility coefficients according to the Central Bureau for Livestock Feeding (CVB, Lelystad, the Netherlands).

^5^Non-starch polysaccharides.

^6^Apparent ileal digestibility, g/kg.

### Sample collection and dissection procedure

Blood samples were collected for serum and plasma at d 7 and dissection days (d28-29) after the morning meal ingestion via the ear-vein^22^. For serum, blood samples were collected in sterile Vacuette tubes containing Z-serum separator clot activator (Greiner Bio-One B.V., Alphen aan den Rijn, the Netherlands), tubes were gently inverted and allowed to clot for at least 30 min. All tubes were centrifuged at 2,200 × *g* for 15 min at 20 °C and serum was extracted. For plasma, blood samples were collected in sterile Vacuette tubes containing lithium-heparin and immediately centrifuged at 3,000 × *g* for 10 min at 4 °C and plasma was extracted. Both serum and plasma were stored at − 80 °C for further analysis on levels of systemic cytokine and chemokines as well as systemic amine metabolite profiles, respectively.

At the dissection days, pigs were anesthetized by injecting pentobarbitone in the ear vein and sacrificed to collect intestinal samples to measure the dietary effects. The small intestine was separated from the stomach and the large intestine. Jejunum and ileum were divided into three equal segments and two sub-segment of the same location of each tissue were sampled for intestinal mucosal layer and resident microbiota. For the intestinal mucosal layer, one of the sub-segment was cut open longitudinally along the lumen and washed with sterile normal saline solution. With a sterile glass slide the mucosal layer was collected, snap frozen in liquid nitrogen and stored at − 80 °C for further analysis of genome-wide gene expression profiling. From another sub-segment, luminal content was collected to perform a community-scale analysis of the gut microbiota.

### Chemical analysis of the diet

Representative samples of dried and ground diets were chemically analyzed for dry matter (DM) (^66^; by four hours drying at 104 °C), sugar^67^, starch^68^, ash (^69^; after three hours ashing at 550 °C), ether extract (^70^; by extraction with petroleum ether) and nitrogen ((N)^71^; by Kjeldahl method and crude protein (CP) calculated as Nx6.25). The results are shown in Table 3.

### Small intestinal genome-wide transcriptome profiling

From each individual pig, jejunal and ileal tissue samples were collected, subsequently total RNA extraction was performed. Total RNA from individual samples from each intestinal tissue was extracted using trizol reagent (Life Technologies, California, United States) as recommended by the manufacturer. Homogenized tissue samples were dissolved in 5 ml of trizol reagent (ThermoFisher Scientific, Lelystad, NL). After centrifugation the supernatant was transferred to a fresh tube. Subsequently a phase separation with chloroform was performed as described by the manufacturer. The RNA was precipitated and dissolved, thereafter quantified by absorbance measurements at 260 nm. Quality check of the RNA samples was performed with the Agilent Bioanalyzer (Agilent, CA, USA). Details of biological replicate per treatment are shown in Supplementary figure S5. One RNA sample from ileum belonging to BSF group failed to pass the QC performed with the Agilent Bioanalyzer. Downstream handling of the samples, i.e. labelling, hybridization, scanning, feature extraction, and QC by statistical analysis was performed as described previously^72^. Briefly, labelling was done as recommended by Agilent Technologies using the One-Color Microarray-Based Gene Expression Analysis Low input Quick Amp Labeling. The input was 200 ng of total RNA and 600 ng of labelled cRNA was used on the 8 pack array. Hybridization was performed as described in the One-Color Microarray-Based Gene Expression Analysis Low input Quick Amp Labeling protocol from Agilent in the hybridization oven (G2545A hybridization Oven Agilent Technologies). The hybridization temperature was 65 °C with rotation speed 10 rpm for 17 h. After 17 h the arrays were washed as described in the One-Color Microarray-Based Gene Expression Analysis Low input Quick Amp Labelling protocol from Agilent. The porcine Agilent microarray slides, G2519F *Sus scrofa* (035,953), harboring 43,803 probes, were used and scanned using the DNA microarray scanner with Surescan high resolution Technology (Agilent Technologies). Agilent Scan Control with resolution of 5 µ, 16 bits and PMT of 100%. Feature extraction was performed using protocol 10.7.3.1 (v10.7) for 1 colour gene expression.

The files generated by the feature extraction software were loaded in the Gene Expression Omnibus from NCBI with the accession number GSE98261, in which a log_2_-transformation and quantile normalization was carried out as described^73^, by executing different packages, including LIMMA^74^ within R. Thereafter, principle component analysis (PCA; unsupervised) was performed using Canoco 5; v5.10^75^. To gain further insight into the biological meaning of the treatment induced changes in the jejunal and ileal transcriptome, two separate analysis approaches were adopted. In the first approach, transcripts were defined as significantly differentially expressed when the log fold-change (FC) between the treatment groups for both intestinal locations was > 1 or <−1 and the adjusted P-value (false discovery rate) of LIMMA was < 0.05. In the second approach, we employed Gene Set Enrichment Analysis (GSEA) on the comparison between BSF and SBM group with no pre-filtering of differentially expressed genes. Gene Set Enrichment Analysis (GSEA) was performed separately for jejunum and ileum. We loaded the normalized intensity values of all annotated genes per treatment and BSF was compared to SBM. The following settings were different from the default settings: (1) permutations were performed on the gene sets and (2) the chip platform was set to gene symbol. Gene Ontology related gene sets of biological processes along with KEGG pathway^76^ related gene sets databases (v5.1) were loaded for analysis. A less stringent cut-off i.e. FDR < 0.1, was set to identify significant differential enriched gene-sets. InteractiVenn^77^ was used to visualize significant GSEA results.

### Small intestinal microbiota profiling

#### Microbial DNA extraction

Details of biological replicate per treatment are shown in Supplementary figure S5. The biological replicates range from 5 to 8 animals per treatment group due to non-availability of luminal digesta. Luminal content of each small intestinal location was mixed 1:1 with PBS and vortexed, spun for 5 min (300 × *g*) at 4 °C. DNA was extracted from small intestinal content by the repeated bead beating method (Yu and Morrison, 2004) using QIAamp PowerFecal DNA Kit (Qiagen, Hilden, Germany) according to manufacturer’s instructions. PowerBead solution (750 μl) was added to the 5 ml Eppendorf tube containing small intestinal digesta approximately 200 mg of luminal content (wet weight) was used for microbial DNA extraction. The quality and quantity of extracted DNA samples were checked by gel electrophoresis (only representative samples) and Nanodrop (Agilent, CA, USA), respectively.

#### 16S rRNA gene based amplicon sequencing

Library construction of the V3 hypervariable region (from 16S rRNA gene) followed by sequencing on an Illumina MiSeq platform (paired end reads; 2*300 bp) were performed. Amplicons of the V3 hypervariable region of the 16S rRNA gene, were generated using the primer set V3_F (CCTACGGGAGGCAGCAG) and reverse primer V3_R (ATTACCGCGGCTGCTGG). Polymerase chain reaction (PCR) conditions were as follows: 2 min at 98 °C, 15 × (10 s at 98 °C, 30 s at 55 °C, and 10 s at 72 °C), and 7 min at 72 °C. The PCR products were mixed with the same volume of 1 × loading buffer (contained SYBR green) and were detected by electrophoresis on 2% agarose gel. Prior to library preparation, the PCR products were mixed in equimolar ratio and purified using Qiagen Gel Extraction Kit (Qiagen, Hilden, Germany). Samples were sequenced by targeted-amplicon 16S sequencing using the MiSeq sequencer (Illumina, San Diego, California, United States). Subsequently, the sample-specific barcodes and primer sequences were trimmed from the Illumina raw reads and the file generated were uploaded in sequence read archive (SRA) from NCBI with the accession number PRJNA669414.

#### 16S based amplicon sequencing data analysis

The trimmed paired end reads were imported into the CLC Genomics Workbench version 20.0.4 and were processed using the CLC Microbial Genomics Module version 2.5.1 (CLC bio, Arhus, Denmark). The paired end reads were merged into one high quality representative sequence using CLC default parameters (Mismatch cost = 1, Minimum score = 40, Gap Cost = 4, Maximum unaligned end mismatches = 5). The sequences were then clustered into operational taxonomic unit (OTUs) at 97% identity threshold, followed by taxonomic annotation using SILVA database v132 (released on Dec 13, 2017)^78^. The OTU table is further filtered by removing OTUs with low abundance (Minimum combined count = 10), to get a final abundance table for each sample. Using the relative abundance of matched OTUs at genus level, principle component analysis (PCA; unsupervised) was performed using Canoco 5 (v5.10) according to software developer’s instructions^75^.

The phylogenetic tree was constructed using Maximum Likelihood Phylogeny tool based on a Multiple Sequence Alignment of the OTU sequences (top 100 most abundant OTUs) generated by MUSCLE (Multiple Sequence Comparison by Log- Expectation) tool^79^ in the workbench. The Maximum Likelihood Phylogeny tool determines the probability of the sequences in the tree, using Neighbor Joining as construction method and Jukes Cantor as Nucleotide substitution model. Abundance analysis, such as alpha- and beta- diversity, PERMANOVA analysis as well as differential abundance analysis (DAA) were performed using CLC- Microbial Genomics Module. Alpha- and beta-diversity measures were calculated with the relative abundance of matched OTU at genus level. To show whether genus abundance profiles of replicate samples for each dietary treatments taken from different locations i.e. jejunum and ileum, varied significantly according to the dietary treatments within a location or not. Comparison of the alpha-diversity measures i.e. Observed species and Shannon indices; taking into account the number of species and the evenness of the species was performed by a Mann Whitney U-test (non-parametric) or Student’s T-test (parametric) in GraphPad Software (8.1.1). Bray–Curtis measure was used to calculate distance matrices for the beta diversity. To visualize the beta diversity results, Principal Coordinate Analysis (PCoA) was performed on the dissimilarity matrices. Additionally, to measure the effect size and significance on beta-diversity (i.e. Bray Curtis) of the replicate digesta samples for each dietary treatments within a location, permutational multivariate analysis of variance (PERMANOVA) analysis, also known as non-parametric MANOVA^80^, was performed using OTU abundance profiles. Furthermore, we focused on the taxonomic distribution of the abundant bacteria derived from the 16S rRNA gene sequences in jejunal and ileal digesta of pigs fed either BSF or SBM based diets. Multivariate redundancy analysis (RDA) was employed to identify microbial signatures (with response score >  ± 0.5) in different dietary treatment groups within each small intestinal location using CANOCO 5. Additionally, the DAA tool of CLC- Microbial Genomics Module was used to characterize the microbial differences at genus level with False Discovery Rate (FDR) < 0.05 with fold change > 5, between the digesta samples for the dietary treatment groups collected from each small intestinal location. Heat maps were constructed by hierarchical clustering of microbial families (selected from DAA) in Perseus software (version 1.6.14.0; available at: http://www.maxquant.org/), where relative abundance values were normalized by z-score transformation. For hierarchical clustering, Euclidean distance was utilized to measure the distance and clustering was conducted using the average linkage method.

### Systemic metabolomics profiling

#### Assay description

The amine profiling was performed as described previously^81^. Briefly, 5 µL of each sample was spiked with an internal standard solution (Supplementary Table S1), thiol amines are released from proteins and converted to a reduced form using Tris-(2-Carboxyethyl)phosphine (TCEP). Then proteins were precipitated by the addition of MeOH. The supernatant was transferred to an Eppendorf tube (Eppendorf, Hamburg, Germany) and dried in a speedvac (Eppendorf, Hamburg, Germany). The residue was reconstituted in borate buffer (pH 8.5) with AQC reagent (Waters, Etten-Leur, The Netherlands). After reaction, the vials were transferred to an autosampler tray (Waters, Etten-Leur, The Netherlands) and cooled to 10 °C prior to injection. For amine metabolite analysis, 1 µL of the reaction mixture was injected into the UPLC-MS/MS system using an Accq-Tag Ultra column (Waters, Etten-Leur, The Netherlands).

#### Equipment

We employed an ACQUITY UPLC system with autosampler (Waters, Etten-Leur, The Netherlands) was coupled online with a Xevo Tandem Quadrupole (TQ) mass spectrometer (Waters, Etten-Leur, The Netherlands) operated using QuanLynx data acquisition software (version 4.1; Waters, Etten-Leur, The Netherlands). The Xevo TQ was used in the positive-ion electrospray mode and all analytes were monitored in multiple reaction monitoring (MRM) using nominal mass resolution.

#### Data processing and quality check (QC) of metabolomics data

Acquired data were evaluated using TargetLynx software (Waters, Etten-Leur, The Netherlands), by integration of assigned MRM peaks and normalization using proper internal standards. For analysis of AA, their 13C15N-labeled analogues were used and for other amines, the closest-eluting internal standard was employed (Supplementary file S3). Blank samples were used to correct for background, and in-house developed algorithms were applied using the pooled quality check (QC) samples to compensate for shifts in the sensitivity of the mass spectrometer over the batch analysis^82^. Out of 76 targeted amine metabolites, we could detect 58 amines that comply with the acceptance criteria of QC corrections. The data are represented as relative response ratios (amine target area/area of internal standard; unit free) of these metabolites (after QC) are available in the Supplementary file S3 (also available online: http://doi.org/10.5281/zenodo.4445165).

#### Metabolomics data analysis

To get more insight into the variability in the amine metabolomics data, PCA was performed in Canoco (V5.0) using the intensities of the identified amine metabolites from plasma sample of pigs fed with either SBM or BSF-based diets (Supplementary file S3). Employing the statistical module of MetaboAnalyst 4.0^83^, the heat map graphic distances were measured using Euclidean distances and the clustering algorithm using a Ward dendrogram. The amine metabolites data was normalized by the pooled sample from group SBM and thereafter log transformation to the data was performed. To retain the most contrasting patterns among the treatment groups, the top 25 metabolites ranked by Student’s T-tests were displayed in the heat-map. Additionally, fold changes of the amine metabolites were calculated using the statistic suits of MetaboAnalyst 4.0. Metabolic pathway analysis module of MetaboAnalyst 4.0^83^ was employed to determine the amine metabolism that was affected by the BSF diet compared to the SBM diets. All the compound names of the metabolites were matched with the human metabolome database (HMDB). To normalize the data, a log transformation was performed. Thereafter, the human pathway library was selected and a reference metabolome (Supplementary file S3) based on our technical platform was uploaded. The analysis includes pathway enrichment analysis and topological analysis. The impact-value threshold calculated from the topology analysis was set at 0.4 and the –log (p)-value calculated from pathway enrichment was set to 5 to identify the most related metabolic pathway^21^.

### Systemic inflammatory marker profiling

Serum cytokine and chemokine concentrations (pg/ml) were measured using a ProcartaPlex Porcine kit (Affymetrix, eBIOscience, Vienna, Austria)^22^. Calibration curves from recombinant cytokine and chemokine standards were prepared for the 8-point standard dilution set with fourfold dilution steps in sterile PBS. The samples were measured using a Bio-Plex MagPix Multiplex Reader (Bio-Rad Laboratories Inc. by the Luminex Corporation, The Netherlands). The Bio-Plex Manager software's five-parameter logistic curve fitting (5PL) method was used for raw data analysis and calculation of cytokine concentrations. Cytokine concentration levels are presented as means ± SEM. Statistical analysis was performed by Student’s T-test to calculate P value (two-tailed) using GraphPad prism (v5.03) for Windows Vista (GraphPad Software, San Diego, California, USA). P value < 0.05 was considered significant.

### Ethics statement

All procedures were approved by the Wageningen Animal Ethics Committee (Wageningen, The Netherlands; accession number 2014099.b) and carried out according to the guidelines of the European Council Directive 86/609/EEC dated November 1986. The reported study is in compliance with the ARRIVE guidelines.

## Supplementary Information

Supplementary Figures and Tables

Supplementary File S1

Supplementary File S2

Supplementary File S3

Supplementary Information

## Data Availability

The microarray data is available in the Gene Expression Omnibus from NCBI with the accession number GSE98261. The microbiome data is available in the Sequence Read Archive from NCBI with the accession number PRJNA669414. The amine metabolite data are available available online: http://doi.org/10.5281/zenodo.4445165 and also in the Supplementary file S1 of this manuscript.
