# Supplementary material for: Local intestinal microbiota response and systemic effects of feeding black soldier fly larvae to replace soybean meal in growing pigs
Source: Sci Rep. 2021 Jul 23;11:15088. doi: 10.1038/s41598-021-94604-8 (PMC8302639; doi:10.1038/s41598-021-94604-8)
Supplement: Supplementary file 1 — Supplementary Figures and Tables [file 41598_2021_94604_MOESM1_ESM.docx]

**Local intestinal microbiota response and systemic effects of feeding black soldier fly larvae to replace soybean meal in growing pigs**

Soumya K. Kar^1*^, Dirkjan Schokker^2^, Amy C. Harms^3,4^, Leo Kruijt^2^, Mari A. Smits^2^_,_ Alfons J. M. Jansman^1^

^1^ Wageningen Livestock Research, Animal Nutrition, Wageningen University & Research, Wageningen, The Netherlands

^2^ Wageningen Livestock Research, Animal Breeding and Genomics, Wageningen University & Research, Wageningen, The Netherlands

^3^Netherlands Metabolomics Centre, Leiden University, Leiden, the Netherlands

^4^Department of Analytical Biosciences, Leiden University, Leiden, the Netherlands

* Correspondence: Dr. Soumya Kanti Kar, [soumya.kar@wur.nl](mailto:soumya.kar@wur.nl)


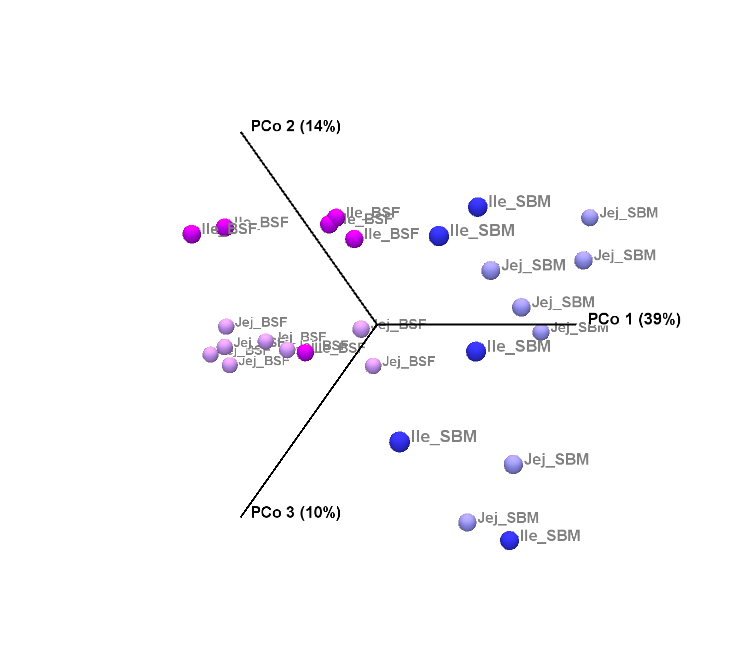


**Supplementary figure S1.** **Beta diversity of intestinal microbiota seen as a 3D Principal Coordinate Analysis (PCoA).** Relative abundance of the microbiome at genus level in jejunum (Jej) and ileum (Ile) digesta of pigs fed soybean meal (SBM) or black soldier fly (BSF) based diets.

**
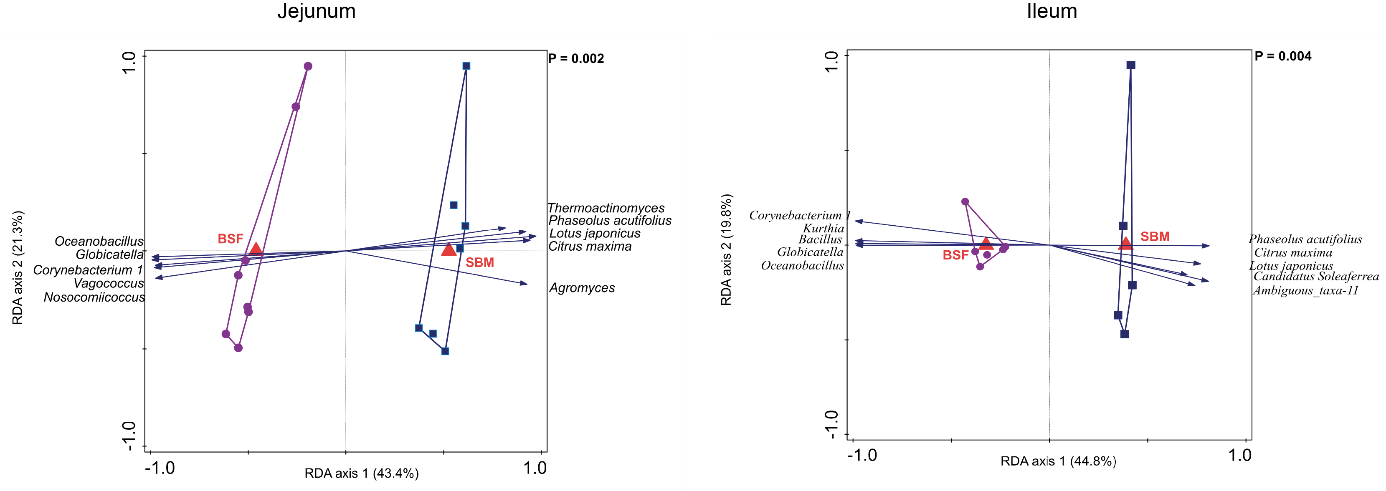
Supplementary figure 2. Redundancy analysis with microbial abundance at genus level in small intestinal digesta of pigs fed a diet with either soybean meal (SBM) or black soldier fly (BSF) as single protein source.** Left panel depicts jejunum, where RDA axis 1 explains 43.4% and RDA axis 2 explains 21.3% of the variation. The right panel depicts ileum, where RDA axis 1 explains 44.8% and RDA axis 2 explains 19.8% of the variation). Samples are depicted by filled purple circles representing the BSF diet or filled blue squares representing the SBM diet, the averages are depicted by red triangles with their corresponding diet also in red.

**
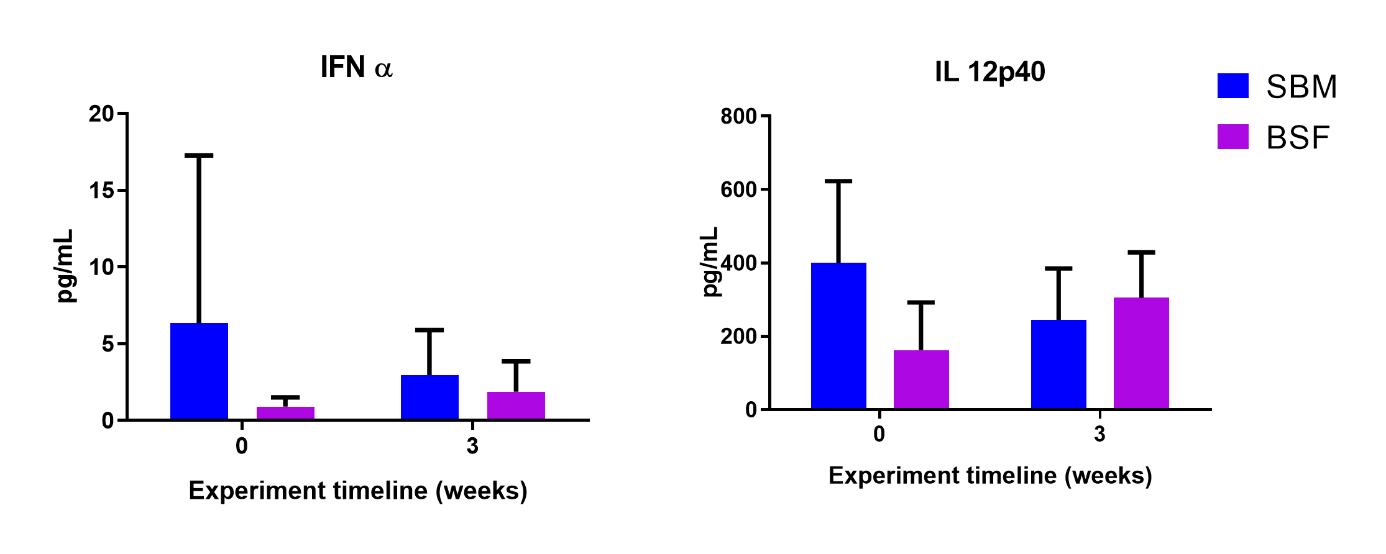
Supplementary figure S3. Cytokine and chemokine concentrations in blood plasma in response to diets with insect protein (BSF) or SBM as dietary protein source.** Bar plots are mean values of the measured level of cytokine and chemokine in the treatment groups (n=8); whiskers are standard error mean. No significant (P<0.05) difference was observed in the experimental diets compared to SBM based diet. SBM, soybean meal; BSF, Black solider fly.

**
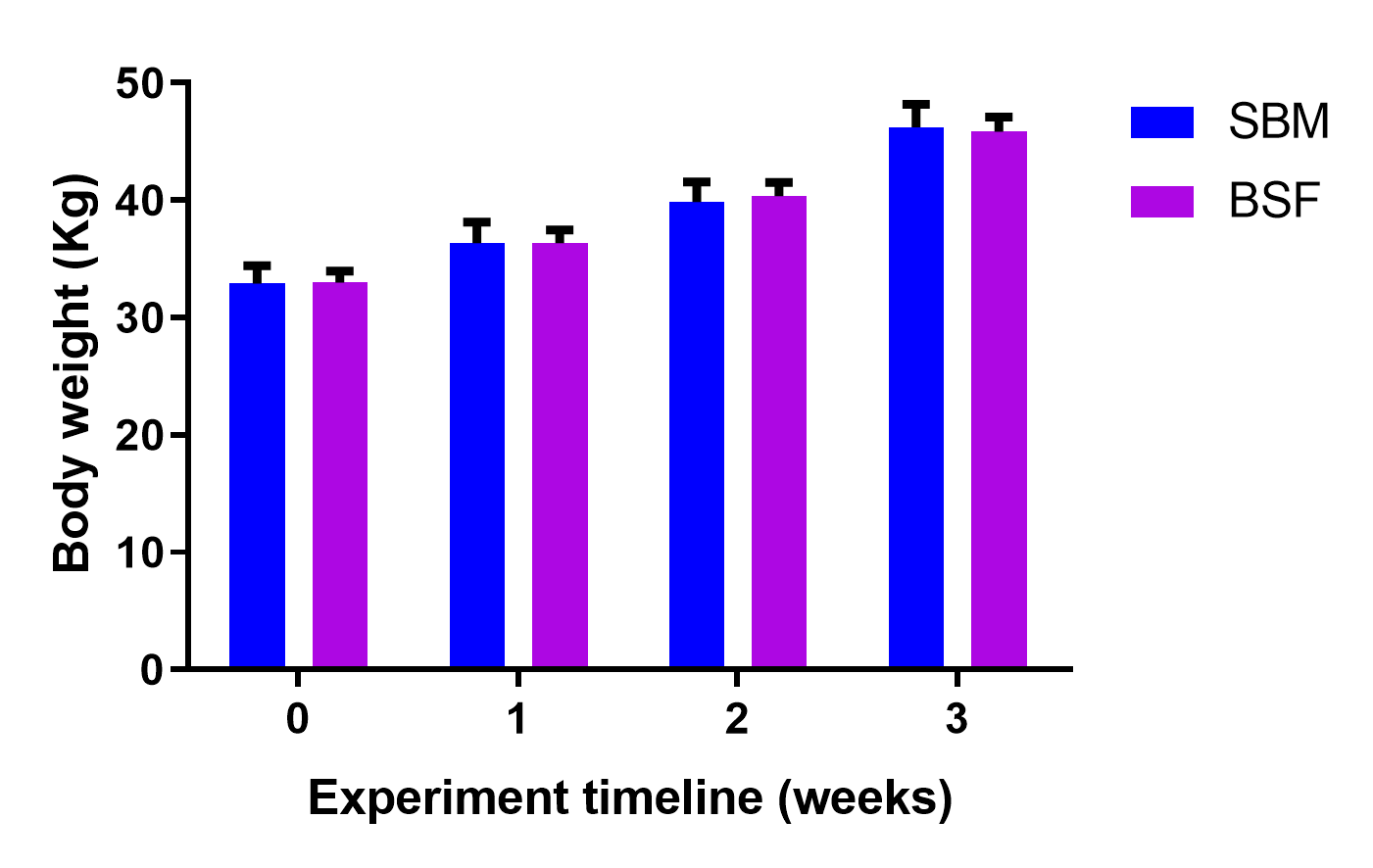
**

**Supplementary figure S4.** **Average body weight in response to diets** **prepared with SBM or BSF as single protein source.** Bar plots are mean values of the body weight (in kg) in the treatment groups (n=8); whiskers are standard error mean. No significant (P < 0.05) difference was observed in the experimental diets compared to SBM based diet. SBM, soybean meal; BSF, Black solider fly.

**
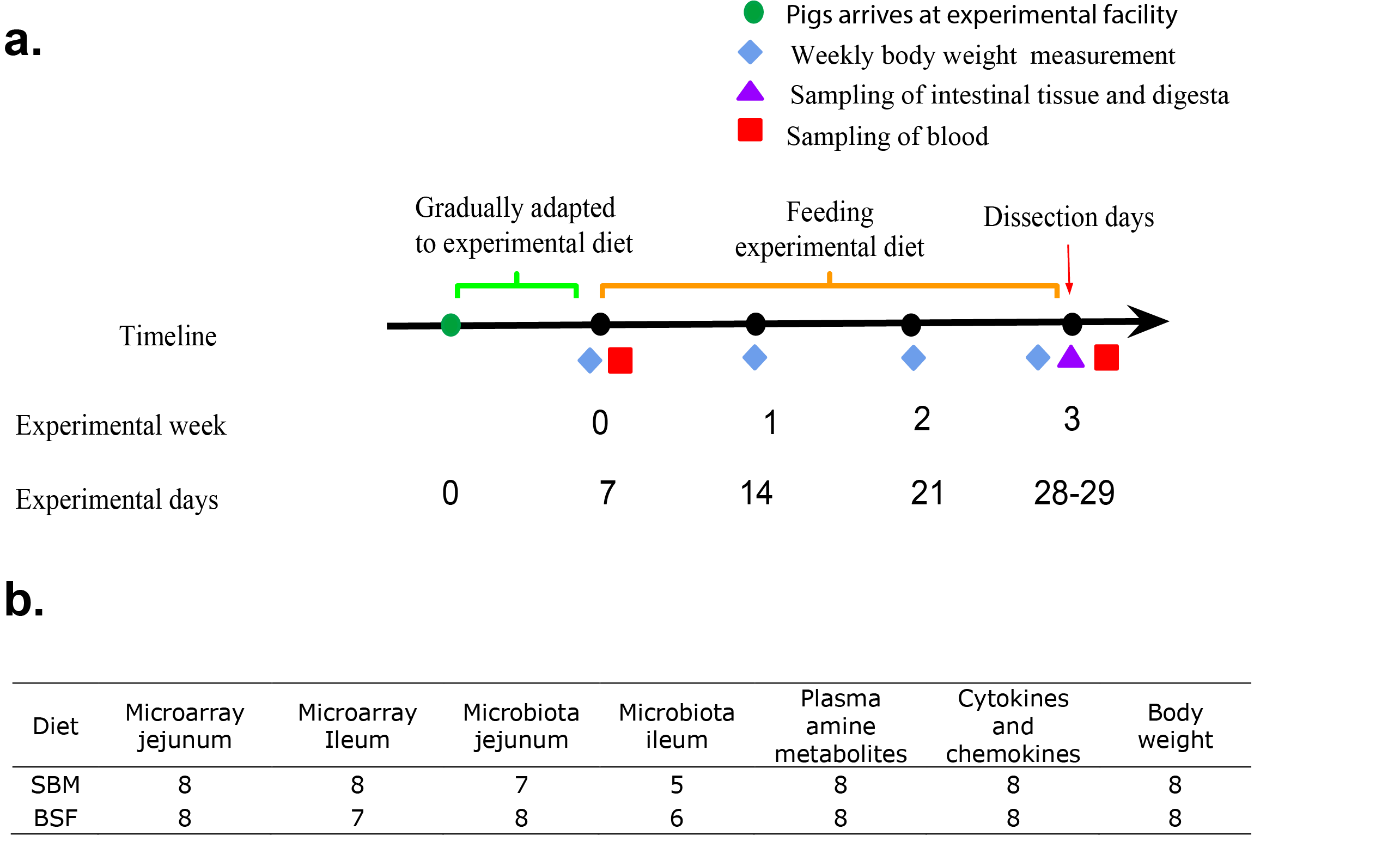
Supplementary figure S5. Schematic representation of the experimental design.** (a) The solid black dot in the timeline represents the corresponding experimental days/age of the pig (weeks). Jejunal and ileal tissue and its digesta were used for transcriptome and microbiota analysis. Blood serum was collected for analysis of systemic immune signaling molecules and blood plasma collected on dissection day was analyzed for systemic amine metabolites. (b) The number of animals (biological replicates) per treatment in respective read-out parameters considered for this study.

**Supplementary table S1. Table with sample information, read, and OTU counts**. Number of reads after quality control and removal of chimeric sequences Number of OTUs at an identity threshold of 97%. SBM, soybean meal; BSF, Black solider fly.

| **Sample** | **Diet** | **Location** | **Total number of reads** | **Reads in OTUs** | **Number of OTUs** |
| --- | --- | --- | --- | --- | --- |
| I15A_TAGGCATG-GCGTAAGA | SBM | Ileum | 438,002 | 354,966 | 516 |
| J20E_CGTACTAG-AAGGAGTA | BSF | Jejunum | 599,160 | 334,287 | 976 |
| I19A_CTCTCTAC-ACTGCATA | SBM | Ileum | 707,455 | 390,245 | 981 |
| J21A_CGTACTAG-GCGTAAGA | SBM | Jejunum | 608,011 | 405,192 | 1400 |
| J28E_AGGCAGAA-GTAAGGAG | BSF | Jejunum | 841,564 | 490,573 | 1534 |
| J3E_TAAGGCGA-TATCCTCT | BSF | Jejunum | 444,095 | 241,863 | 1109 |
| I22E_CTCTCTAC-AGAGTAGA | BSF | Ileum | 561,539 | 348,458 | 843 |
| J12E_CGTACTAG-ACTGCATA | BSF | Jejunum | 520,804 | 276,556 | 926 |
| I9E_TAGGCATG-TATCCTCT | BSF | Ileum | 334,667 | 216,817 | 832 |
| I28E_CAGAGAGG-ACTGCATA | BSF | Ileum | 479,381 | 250,771 | 1031 |
| J44E_GGACTCCT-CTCTCTAT | BSF | Jejunum | 497,480 | 261,455 | 967 |
| J39A_TCCTGAGC-AAGGAGTA | SBM | Jejunum | 468,835 | 341,179 | 929 |
| J2A_TAAGGCGA-CTCTCTAT | SBM | Jejunum | 496,854 | 346,390 | 1235 |
| I21A_CTCTCTAC-TATCCTCT | SBM | Ileum | 510,098 | 287,637 | 792 |
| J33E_TCCTGAGC-ACTGCATA | BSF | Jejunum | 624,328 | 402,230 | 1779 |
| J15A_CGTACTAG-CTCTCTAT | SBM | Jejunum | 572,887 | 411,952 | 932 |
| J42A_GGACTCCT-ACTGCATA | SBM | Jejunum | 592,991 | 451,847 | 1273 |
| J9E_TAAGGCGA-GCGTAAGA | BSF | Jejunum | 541,928 | 285,271 | 1076 |
| J19A_CGTACTAG-GTAAGGAG | SBM | Jejunum | 651,260 | 434,419 | 1374 |
| I44E_GCTACGCT-CTCTCTAT | BSF | Ileum | 603,518 | 348,273 | 965 |
| I20E_CTCTCTAC-CTCTCTAT | BSF | Ileum | 343,837 | 186,720 | 667 |
| J22E_CGTACTAG-CTAAGCCT | BSF | Jejunum | 582,332 | 315,315 | 1273 |
| J29A_AGGCAGAA-AAGGAGTA | SBM | Jejunum | 875,336 | 668,299 | 1886 |
| I2A_GGACTCCT-AAGGAGTA | SBM | Ileum | 981,218 | 714,818 | 1041 |
| I3E_GGACTCCT-GCGTAAGA | BSF | Ileum | 322,523 | 215,476 | 733 |
| I39A_CAGAGAGG-GCGTAAGA | SBM | Ileum | 783,424 | 595,225 | 1298 |

**Supplementary table S2. Transcriptomics response in the jejunal and ileal mucosa of pig fed diets with BSF relative to a diet with SBM**

| **Tissue** | **Biological processes and pathways** | |
| --- | --- | --- |
|  | **Up-regulated (6)** | **Down-regulated (188)** |
| **Jejunum** | KEGG metabolism of xenobiotics by cytochrome p450 | KEGG ECM receptor interaction |
|  |  | KEGG focal adhesion |
|  |  | Potassium ion transport |
|  |  | Generation of neurons |
|  |  | KEGG ribosome |
|  |  | Neurite development |
|  |  | Neurogenesis |
|  |  | Cell-cell adhesion |
|  |  | Neuron development |
|  |  | Neuron differentiation |
|  |  | Synaptic transmission |
|  | **Up-regulated (31)** | **Down-regulated (55)** |
| **Ileum** | KEGG tryptophan metabolism  KEGG PPAR signalling pathway | KEGG ECM receptor interaction |
|  |  | Neuron differentiation |
|  |  | Generation of neurons |
|  |  | Neurogenesis |
|  |  | Neurite development |
|  |  | Neuron development |
|  |  | Axonogenesis |
|  |  | Negative regulation of cellular protein metabolic process |

Differential gene-sets enriched in Gene Set Enrichment Analysis (GSEA) corresponds to the biological processes and pathways. The number within parenthesis represents the core enriched genes (full list in File S2) within the differential enriched gene-sets in GSEA.

**Supplementary table S3. T-test and fold changes values of differentially abundant amine metabolites increased in blood plasma of pigs fed a BSF diet compared to a SBM diet, based upon the results of heatmap and metabolic pathway analysis.**

| **Amine metabolites** | **P-value** | **FDR** | **Fold change** |
| --- | --- | --- | --- |
| Sarcosine | 6.67E-08 | 6.45E-07 | 5.6 |
| Homocitrulline | 2.65E-06 | 1.92E-05 | 5.5 |
| L-Alpha-aminobutyric acid | 5.71E-10 | 1.10E-08 | 5.0 |
| L-Methionine | 5.60E-09 | 7.39E-08 | 2.8 |
| S-Methylcysteine | 2.57E-11 | 1.49E-09 | 2.6 |
| O-Acetyl-L-serine | 6.37E-09 | 7.39E-08 | 2.3 |
| L-Valine | 1.30E-10 | 3.78E-09 | 2.1 |
| L-Methionine sulfoxide | 1.30E-06 | 1.07E-05 | 2.1 |
| Taurine | 0.0011426 | 0.0047336 | 1.6 |
