# Supplementary material for: Local intestinal microbiota response and systemic effects of feeding black soldier fly larvae to replace soybean meal in growing pigs
Source: Sci Rep. 2021 Jul 23;11:15088. doi: 10.1038/s41598-021-94604-8 (PMC8302639; doi:10.1038/s41598-021-94604-8)
Supplement: Supplementary file 5 — Supplementary Information [file 41598_2021_94604_MOESM5_ESM.docx]

**Local intestinal microbiota response and systemic effects of feeding black soldier fly larvae to replace soybean meal in growing pigs**

Soumya K. Kar^1*^, Dirkjan Schokker^2^, Amy C. Harms^3,4^, Leo Kruijt^2^, Mari A. Smits^2^_,_ Alfons J. M. Jansman^1^

^1^ Wageningen Livestock Research, Animal Nutrition, Wageningen University & Research, Wageningen, The Netherlands

^2^ Wageningen Livestock Research, Animal Breeding and Genomics, Wageningen University & Research, Wageningen, The Netherlands

^3^Netherlands Metabolomics Centre, Leiden University, Leiden, the Netherlands

^4^Department of Analytical Biosciences, Leiden University, Leiden, the Netherlands

* Correspondence: Dr. Soumya Kanti Kar, [soumya.kar@wur.nl](mailto:soumya.kar@wur.nl)

**Supplementary file S1**: Relative abundance and list of identified bacterial genera by differential abundance analysis of microbiome data.

**Supplementary file S2**: The list core enriched gene in the biological processes and pathways of the enriched gene sets as shown in supplementary table S2.

**Supplementary file S3**: Internal standards, detected amine metabolites and used reference metabolome data base for metabolic pathway analysis.
